# Modeling COVID-19 Vaccine Adverse Effects with a Visualized Knowledge Graph Database

**DOI:** 10.3390/healthcare10081419

**Published:** 2022-07-29

**Authors:** Zhiyuan Liu, Ximing Gao, Chenyu Li

**Affiliations:** 1 Stanford Center for Professional Development, Stanford University, Stanford, CA 94305, USA; leonzhiyuan@gmail.com (Z.L.); eleanorgao9526@gmail.com (X.G.); 2 Department of Biomedical Data Science, Stanford University, Stanford, CA 94305, USA

**Keywords:** COVID-19 vaccine, adverse effects, knowledge graph database modeling, graph representational learning, graph embeddings

## Abstract

In this study, we utilized ontology and machine learning methods to analyze the current results on vaccine adverse events. With the VAERS (Vaccine Adverse Event Reporting System) Database, the side effects of COVID-19 vaccines are summarized, and a relational/graph database was implemented for further applications and analysis. The adverse effects of COVID-19 vaccines up to March 2022 were utilized in the study. With the built network of the adverse effects of COVID-19 vaccines, the API can help provide a visualized interface for patients, healthcare providers and healthcare officers to quickly find the information of a certain patient and the potential relationships of side effects of a certain vaccine. In the meantime, the model was further applied to predict the key feature symptoms that contribute to hospitalization and treatment following receipt of a COVID-19 vaccine and the performance was evaluated with a confusion matrix method. Overall, our study built a user-friendly visualized interface of the side effects of vaccines and provided insight on potential adverse effects with ontology and machine learning approaches. The interface and methods can be expanded to all FDA (Food and Drug Administration)-approved vaccines.

## 1. Introduction

Adverse effects have been an important issue of vaccines, which are tightly monitored and evaluated each year [1]. In December 2020, the FDA issued the Emergency Use Authorization EUA for two mRNA-based COVID-19 vaccines (BNT162b2 from Pfizer-BioNTech and mRNA-1273 from Moderna) as 2-dose series and in February 2021, the FDA issued another EUA for one viral-based COVID-19 vaccine (JNJ-78436735 from Johnson & Johnson). Since then, the local and systemic adverse reactions after receipt of these COVID-19 vaccines have been summarized and studied across the world. WHO revised the regulation for safety and effectiveness in May 2022 to help healthcare professionals in the explanation of the oversight of COVID-19 vaccines [2]. Across the European Union (EU), a suitable pharmacovigilance system has to be in place to gather and report data on the adverse reactions during the COVID-19 vaccine campaigns [3,4,5]. In the United States, VAERS functions as the reporting and monitoring system to summarize the adverse events of COVID-19 vaccines. Until July 2022 in the US, 599 million doses of COVID-19 vaccines were administered. Though COVID-19 vaccines have been proven effective in preventing complications of COVID-19, including pneumonia, acute respiratory distress syndrome (ARDS), multi-organ failure, septic shock and death, 12,775 preliminary reports of death (0.0023%) among people who received a COVID-19 vaccine were reported at VAERS [6]. Adverse effects after COVID-19 vaccination include but are not limited to fatigue, muscle pain, headache, chills, injection site reaction, joint pain, fever, sore throat and allergic reaction. Allergic reaction or anaphylaxis was reported in 0.2% of participants after full vaccination [7]. Though serious adverse effects are rare, the general adverse effects cannot be overseen [8,9]. This indicates continued monitoring and assessment of adverse effects of COVID-19 vaccines are required to further improve our current understanding of safety and decision-making in the implementation of vaccination.

Some previous works focused on the prediction of vaccine outcomes and adverse effects [10,11]. Gonzalez-Dia’s group has provided a machine learning method for data processing, feature selection, algorithm selection and testing for the prediction of vaccine-induced immunity [12]. The other group conducted the classification of post-COVID-19 vaccination reactogenicity with decision tree and random forest methods to find the features that lead to hospitalization and patient death [13]. One previous approach utilized an ontology method to support operable COVID-19 context for automatic governance of bioethics processes [14]. While efforts are made on the prediction of COVID-19 vaccine side effects, a visualized interphase is still largely required for healthcare providers, patients, and healthcare officers to easily access the information. Therefore, our goal is to utilize ontology and machine learning methods to analyze COVID-19 vaccine adverse events and build a visualized system to provide insight into the adverse events data and the analyzed results.

In this study, we took the use of the VAERS data of COVID-19 vaccines to first build a visualized interface with user-friendly approaches for healthcare providers, patients, vaccine manufacturers and government officers. While some security concerns and suggestions have been proposed for storing COVID-19 patient data in different NoSQL DBMSs (database management systems) [15], we used graph database neo4j [16] to store the processed data for our system, as it is open-sourced and widely used in software with complex relationships. Further, no security concerns exist given patient data is de-identified in VAERS. Ontology was utilized to set the nodes and relations in the network with neo4j software [16]. In the second part of this study, we demonstrated several applications to conduct analysis with our proposed system, including querying, finding similarities and classification. Some previous work has focused on leveraging machine learning models on VAERS data to predict death risk [17]. We also utilized this ontology network and the information on the adverse effects to build a machine learning model for the prediction of key feature symptoms that lead to hospitalization and treatments after COVID-19 vaccination. A node2vec method [18] was utilized to predict the most predictive symptoms related to hospitalization outcomes of patients, and the model was evaluated with a confusion matrix method. The probability of a patient’s need for hospitalization was provided by the final model.

## 2. Materials and Methods

### 2.1. Dataset

Three datasets (VAERSDATA, VAERSSYMPTOMS, VAERSVAX) are extracted from VAERS [1], which is an open-source national reporting system for safety problems of licensed vaccines (Supplementary Table S1). The total vaccination data in the US was extracted from VAERS from 2021–2022 (up to March 2022), and the data was filtered with adverse events associated with vaccinations (majority of the data are COVID-19 vaccines Pfizer-BioNTech, Moderna, Janssen since the pandemic). The unique VAERS ID was used per patient to identify multiple events of a single person. The variables, including age, sex, vaccine manufacture, current illness, disability status, medication usage, allergic history, pre-existing conditions, etc., were extracted and encoded to be utilized in the knowledge graph.

VAERSDATA includes the information of each patient, including patient information, hospitalization, lab results, treatments, vaccination records and allergies (‘VAERS_ID’, ‘RECVDATE’, ‘STATE’, ‘AGE_YRS’, ‘CAGE_YR’, ‘CAGE_MO’, ‘SEX’, ‘RPT_DATE’, ‘SYMPTOM_TEXT’, ‘DIED’, ‘DATEDIED’, ‘L_THREAT’, ‘ER_VISIT’, ‘HOSPITAL’, ‘HOSPDAYS’, ‘X_STAY’, ‘DISABLE’, ‘RECOVD’, ‘VAX_DATE’, ‘ONSET_DATE’, ‘NUMDAYS’, ‘LAB_DATA’, ‘V_ADMINBY’, ‘V_FUNDBY’, ‘OTHER_MEDS’, ‘CUR_ILL’, ‘HISTORY’, ‘PRIOR_VAX’, ‘SPLTTYPE’, ‘FORM_VERS’, ‘TODAYS_DATE’, ‘BIRTH_DEFECT’, ‘OFC_VISIT’, ‘ER_ED_VISIT’, ‘ALLERGIES’). VAERSSYMPTOMS includes the information of symptoms of each patient that takes a vaccine. The VAERSVAX dataset includes the vaccination information, including lot, type, site and name. The full information can be found in Supplementary Tables S2 and S3.

### 2.2. System Overview

**Figure 1:**
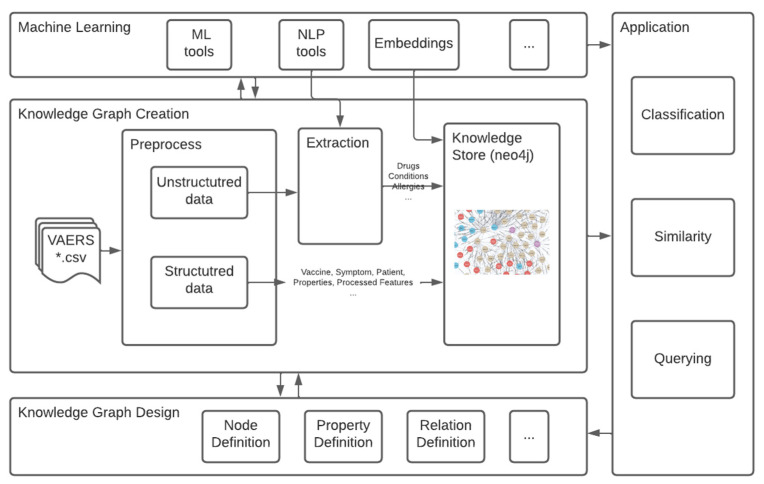
Knowledge Graph-Based System. (The VAERS data includes three dataset (VAERSDATA, VAERSSYMPTOMS, VAERSVAX) in separate spreadsheets, represented as *.csv.).

A knowledge graph-based system to store and analyze the data was developed. The four major components of our system are shown in Figure 1: knowledge graph design, knowledge graph creation, machine learning and application components.

The knowledge graph was designed based on the VAERS data structure and potential use cases from the application. In knowledge graph creation, we preprocess the data, both structured and unstructured, to create knowledge graph nodes, properties and relationships and store them in the neo4j GraphDB. There is a machine learning component to help with the whole process, for example, to extract entities from unstructured data, compute node embeddings and build machine learning models for the application, etc.

With the vaccine side effect knowledge stored, three tasks were applied for application: (1) querying: use cypher query to search the database; (2) similarity: find similarity among the nodes with the help of node embeddings; and (3) classification: classify the needs of hospitalized vaccinated patients with side effects using machine learning methods. We will cover these components in detail in the following sections.

### 2.3. Data Preprocessing

For computation purposes, we sampled ~10 k side effect records. In the future, the methodology can be applied to use all the data. The related nodes are created, including three types: vaccine nodes, symptom nodes and patient nodes. The information on vaccines and symptoms are added as edges to the vaccine nodes and patient symptom nodes. For the unstructured text data, including OTHER_MEDS, CUR_ILL, HISTORY and SYMPTOM_TEXT field, we first cleaned the texts for each node to remove the invalid texts, including “na”, “none”, “unknown”, “n/a”, “no”, “nothing”, “non3”, “unk”, “no.”, “none.”, etc., using the regex matches with lower case since we found those texts occur with high frequencies in those fields and provide no information.

To process the allergies, disease and medical history data, we used NLP (Natural Language Processing) methods to extract the structured entities and add them to our knowledge graph. Specifically, an open-source pretrained medical NER (Named-entity recognition) model was applied. We used a pre-trained scispacy “en_core_sci_md” in the Python ScispaCy [19] package model to link the texts to entities. In this part, RxNorm and Unified Medical Language System (UMLS) ontologies were utilized to be linked with NLP methods. The information from the OTHER_MEDS field was further extracted using the RxNorm linker with UMLS CUI. Historical condition relations are extracted using the UMLS linker from CUR_ILL, HISTORY and SYMPTOM_TEXT with restrictions to only keep disease/symptom entities because of the noisy text information. The disease/symptom entities are determined by the semantic type TUI T047 in UMLS. The allergy histories are also extracted from ALLERGIES fields using the UMLS linker.

### 2.4. Knowledge Graph

The dynamic knowledge graph is processed with neo4j software for the user interface. A free version of neo4j AuraDB was utilized, which is a cloud-based GraphDB, to collaborate on building and exploring the graph data, and this allows 50,000 nodes as a maximum. Though this limits the full nodes to be processed, we managed to evenly split the nodes across the timeline to better present the overall performance of the model. Both the nodes and edges processed in the previous step are imported into the neo4j, and a dynamic interaction user interface was built. See the abstraction of node and edge details in Supplementary Table S4.

Besides the properties shown in Supplementary Table S4, we also store the additional preprocessed features in the knowledge graph as properties. For example, “feature_STATE” includes “STATE” features of patients with the replacement of NA instances to “UNKNOWN”, so the nodes without a “STATE” property will have “feature_STATE” “UNKNOWN”. These features are used for downstream applications, for example, building machine learning models, and they are dynamic features, so they can be updated frequently based on the use cases.

We also compute node2vec embeddings (Aditya Grover, Jure Leskovec) for each node in the graph with the help of Neo4j built-in Graph Data Science Library and calculate the top cosine similarities among the nodes. The embeddings are also saved as a property of each node, and the cosine similarity edges are created. The embeddings and similarity edges are also dynamic and can be updated as frequently as used. We experimented with node embeddings with 300 dimensions and with a parameter return factor P: 0.25 and input factor Q: 4 to make the random walk more concentrated.

### 2.5. Application

With the knowledge graph, we came up with three types of applications: querying, similarity and classification. For querying, we analyzed the statistics about COVID-19 vaccine, symptoms and historical drug/condition/allergy connections by searching in the GraphDB we built using the cypher queries. For similarity, after we computed the node embeddings and top cosine similarities of each node, we also used cypher queries to explore the similarity patterns among the COVID-19 vaccine and other entities.

For classification, the performance of prediction of hospitalization of a patient after receipt of a COVID-19 vaccine with certain symptoms was tested. We use cypher to pull the data from the GraphDB and split the data as 80% training set and 20% test set. In the training set, there are 7354 records without hospitalization (label 0) and 638 with hospitalization (label 1). In the test set, there are 1846 records without hospitalization (label 0) and 152 with hospitalization (label 1). Given the small size of the data with imbalanced labels, the final performance of the model is also evaluated with a confusion matrix, f1 score and precision recall AUC.

We have experimented with several model architectures, including logistic regression and boosted trees. We use grid search methods with cross-validation to find the best parameters tuning on the positive label f1 score. All the models use patient features, including: “SEX”, “STATE”, “AGE_YRS” and “NUMDAYS”. Features “SEX” and “STATE” is one hot encoded, and “AGE_YRS” and “NUMDAYS” are standardized. In addition, features including symptom features, embedding features and combined features were added (Table 1).

## 3. Results

### 3.1. Preprocessing of the Data to Generate Knowledge Graph

The three datasets in 2021 and 2022 on the VAERS database were extracted and preprocessed using python to generate the knowledge graph. In total, 13,670 vaccination side-effect records were extracted, and the top distributions based on the types of the vaccines in the data are presented in Table 2. This is not the final representation in the graph since one patient may have multiple side effects records, but will give some insights into the source data distribution after preprocessing.

From the analysis, COVID-19 side effects are the top one from these two years’ reports, which is over 50 times the rest of the vaccines. Though this may be partially due to the high number of COVID-19 vaccination, it also indicates the importance of the studies on the adverse effect of COVID-19 vaccines.

In the next step, we deciphered different vaccines using the VAX_NAME. The results are consistent with the previous findings that the top three vaccines are the COVID-19 vaccines from Pfizer, Moderna and Janssen, with counts of adverse effects around 50–20 times higher than the rest of the vaccines (Table 3). From the results, the counts may also partially correlate with the number of the vaccination, as Pfizer and Moderna’s side effects reports are higher than Janssen’s, while in recent reports, more adverse effects are found in the Janssen COVID-19 vaccine.

We also analyzed the side effects of vaccines based on different vaccine manufacturers. As shown in Table 4, the results also pointed to the top three COVID-19 manufacturers, which indicates a high number of COVID-19 vaccinations, while the study and analysis of these side effects are largely required.

Furthermore, the top symptoms of the side effects are shown in Table 5, Table 6, Table 7 and Table 8. Common side effects are identified, including headache, fatigue, pyrexia, etc. (Table 5). The treatments are also summarized in Table 6, which indicates the top common drugs used to treat adverse effects. In the meantime, we also analyzed the most common diseases and allergies in the reports (Table 7 and Table 8). COVID-19 is the second highest disease, which is slightly less than hypertensive disease. This indicates the effectiveness of COVID-19 vaccines and side effects should be tightly monitored.

### 3.2. Creation of the GraphDB with Dynamic Interface

In the second part of the study, we utilized the preprocessed dataset to build a user-friendly visualized knowledge graph database. Due to the limitation of the free version on the nodes (50,000) in neo4j software, we focused on the COVID-19 adverse effects records in 2021 and 2022 and randomly selected 5,000 records for the presentation. In the real case with the updated version of neo4j, this method can be applied to present all the adverse effects of FDA-approved vaccines.

**Figure 2:**
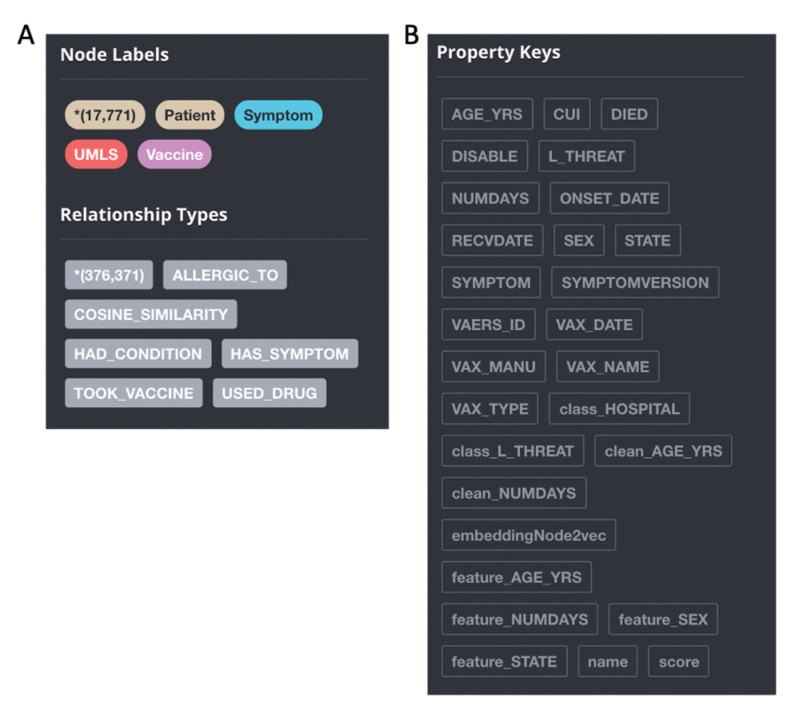
Creation of GraphDB. ((**A**): the summary of nodes and relationships; (**B**): the properties of the knowledge graph in the neo4j interface. The total number of nodes and relationship types are represented in the bracket with “*”.).

As shown in Figure 2A, four types of node labels are built in the GraphDB (patient, symptom, UMLS and vaccine). Each patient and vaccine is set as an individual node. The reports are extracted, and the information is built into the graph as edges, which includes allergic_to, consine_similarity, had_condition, has_symptom, took_vaccine, used_drug, etc. The property keys are set based on the patient information, vaccination information, hospitalization record, symptoms and treatment information in the dataset. This includes age_yrs, CUI, died, diable, L_threat, numdays, onset_date, recvdate, sex, state for each patient node, symptom and symptomversion for each Symptom node, vaers_id, vax_date, vax_type, vax_manu and vax_name, etc. for each Vaccine node. All the nodes are assigned a name property as the name to be displayed in the neo4j interface. For Patient Nodes, their names are the patient ids, and for Symptom and Vaccine Nodes, their name properties are the same with symptom and vax_name properties (Figure 2B). Moreover, we also kept the labels and features as dynamic properties for machine learning modeling, including binary label class_HOSPITAL, node embedding embeddingNode2vec, and processed features, such as one hot encoded feature_SEX, feature_STATE, etc.

**Figure 3:**
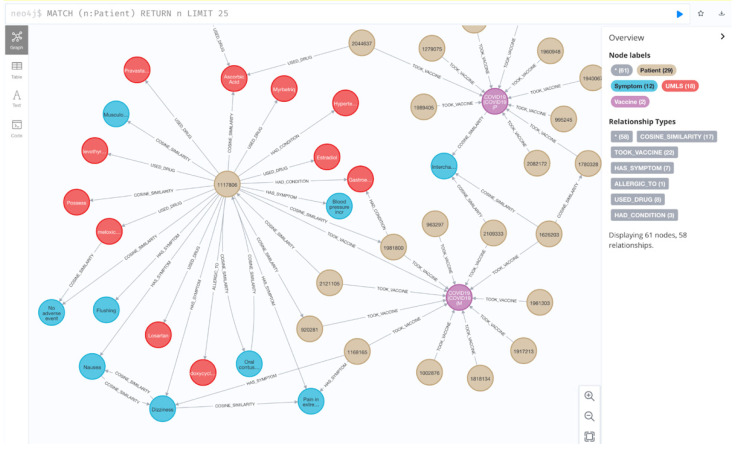
User-friendly dynamic interface of the GraphDB. (The total number of the nodes and relationships is represented in the bracket started with “*”).

The graph database was built based on the previously discussed settings. As shown in Figure 3, the interface is user-friendly, and the record of each patient and each vaccine can be easily visualized. In this example, it shows the subgraph of Patient 1117806 (Patient node in the center of the graph), who has been reported to have taken PFIZER\BIONTECH COVID-19 Vaccine (Vaccine node in purple) and developed Symptoms, including Blood Pressure Increased, Flushing, Nausea, Dizziness and Pain in Extremity (Blue Symptom nodes with HAS_SYMPTOM edges to that patient). In the meantime, the connections and correlations can be directly monitored with the neo4j database. This improves the version of the current dataset on VAERS and other databases. Compared with the traditional datasets, with this approach, all the adverse effects records of vaccines can be imported into a dynamic visualized graph database, and the relationships of each record can be directly monitored.

### 3.3. Application of the GraphDB for Prediction

Besides the advantage of a user-friendly visualized interface and the presentation of all the records in one platform, the graph database built with neo4j can be directly utilized to perform searching, data analysis, train machine learning or deep learning models. In the following part, we tested one application using the graph database built with COVID-19 vaccine adverse effects data.

#### 3.3.1. Querying

With the vaccine adverse effect knowledge graph in hand, we can use the cypher query to search for the connections between vaccines and other entities easily. Some example results from the cypher query are shown in Table 9, Table 10, Table 11 and Table 12.

#### 3.3.2. Similarity

Using the trained node2vec embeddings, we can explore the knowledge graph by finding potential connections about vaccines via the similarity score. Since we have saved both embeddings and the top cosine similarities in the GraphDB, we can also query the similarity result on the fly. Table 13 and Table 14 show some similarity results on COVID-19 vaccines.

#### 3.3.3. Classification

A machine learning model was trained with the graph database to predict the potential hospitalization and death of a patient that received a COVID-19 vaccine and showed certain symptoms. The model results are shown in Table 15.

From the table, we see models trained on embedding only features that do not give us a good result. Our hypothesis is that the embeddings do not learn useful information for this task. Actually, the embedding method is an unsupervised model, so the learned representations cannot be directly applied to the downstream tasks. To improve the results, further experiments can be explored by using embeddings as the first layer of the representations and training neural network-based models on labels so they can be fine-tuned toward the downstream task.

### 3.4. Evaluation of the Prediction Model

**Figure 4:**
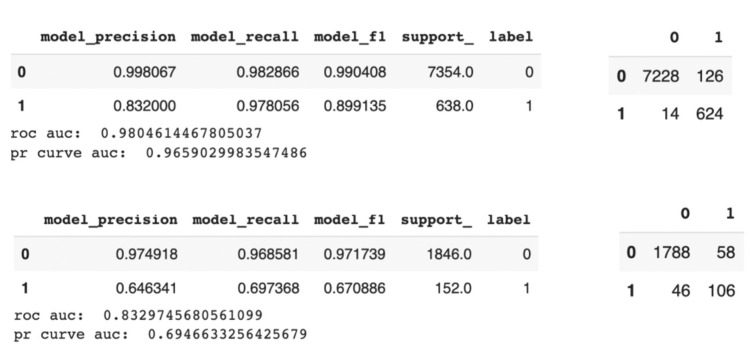
Evaluation results of the model. ((**Top left**): Training metrics. (**Top right**): Training confusion metrics. (**Bottom left**): Test metrics. (**Bottom right**): Test confusion metrics).

To evaluate the performance of the prediction on hospitalization with symptoms of patients after receipt of COVID-19 vaccines, the data were randomly split into training and testing groups (Figure 4). The prediction of the testing group was performed with the trained model with the training batch, and the results were compared with the real outputs of the testing batch. With this approach, the f1 score was utilized to present the performance of the evaluation since the f1 score can take both the precision and recall into consideration and, in this case, both precision and recall are important. From the results, our model on the prediction of hospitalization with symptoms has an f1 score of 0.67 in the testing group for positive predictions. The f1 scores of Eysha Saad’s death risk prediction models on VAERS origin data are between 0.64 and 0.7 using CNN, LSTM and BiLSTM [17]. Though our model predicts the hospitalization task, the simple XGBoost model we built can reach a similar level of accuracy. This indicates that the prediction model using the knowledge graph built can be useful in the analysis of the potential hospitalization and death rate of the patient after receipt of the COVID-19 vaccines.

## 4. Discussion

Our study provides a general method to present all the recorded side effects of vaccines approved by the FDA. One previous study hosted on the Eureka Research Platform collected data from 19,586 registered participants from 26 March 2020 to 19 May 2021 and reported the percentage rate of COVID-19 vaccine adverse effects by participant’s characteristics [7]. While in our study, we proposed a knowledge graph-based system to analyze COVID-19 vaccine side effects. With the user-friendly interface, the users can visualize the side effects and data through the built-in interface.

In addition, we demonstrated examples of building machine learning models to predict hospitalization after COVID-19 vaccination using the system. To model the relationships among the nodes, we calculated node2vec network embeddings for all of the nodes. Node2vec is a random walk-based method that is able to capture higher-order relationships. However, it does not differentiate between node or relationship types. To improve this, there are heterogeneous network embeddings and embeddings over temporal graphs that could be used as better knowledge graph representations [20,21].

However, with limited datasets, in order to better evaluate the model, the real data of patients should be introduced into the system and compared with the suggestions from healthcare providers to tackle the goal of making suggestions on the vaccination of a certain patient. A study in Malaysia established the incidence of adverse COVID-19 drug effects based on the data provided by Sungai Buloh Hospital [22]. This indicates the potential of machine learning models in supporting COVID-19 treatments in the future; for example, in this project, due to limited resources to get suggestions from healthcare experts. As a proposal for the evaluation step, it should be more precise than the precision, recall and f1 score are evaluated with the prediction of the model and the doctor’s suggestions for those patients.

Even though COVID-19 vaccines can effectively reduce serious illness, hospitalization and death rate, there are still vaccine-hesitant patients who will not take vaccines for themselves or their families and the low rate of COVID-19 vaccines acceptability was observed in some other countries [23,24]. The public acceptability rate was reported only at 37.4% in Jordan [23]. With the machine learning model built, a better understanding of the safety of vaccines could be provided; further prediction can be performed with the chance of patients getting adverse effects after taking the vaccine, and provide the corresponding recommendations to the patients or the parents on whether themselves or their children should take the vaccine or not.

Another part of the future work is to improve the automatic importation of adverse effects into the interface. In our case, COVID-19 vaccines reported findings quickly and with software engineering methods, and the model can report the most updated analysis results with the up-to-date data. Further, if more data from the patients are available, for instance, with access to more medical history data, we can infer the relationship between medical history and vaccine adverse effects. This can be achieved by crosslinking different databases using ontologies. With the machine learning model built, further prediction can be performed with the chance of patients getting vaccine adverse effects after taking the vaccines and provide the corresponding recommendations to the patients on whether they should take the vaccine or not.

In summary, our study provided a general method to summarize and present COVID-19 vaccine adverse events using a knowledge graph. This enables patients, healthcare professionals and government officials to quickly visualize the side effects. Though the study may contribute to better regulation and monitoring of COVID-19 vaccines, due to limited resources and datasets, future work is required to correlate medical history and vaccine adverse effects with the crosslinking of different databases using ontologies.

## Figures and Tables

**Table 1:** Classification model structure and features.

Model ID	Model Type	Additional Features
1	Logistic Regression	Symptoms
2	XGBoost	Symptoms
3	Logistic Regression	Embedding
4	XGBoost	Embedding
5	XGBoost	Symptoms + Embedding

**Table 2:** Distributions of vaccine types.

VAX_TYPE	Counts
COVID-19	12,834
VARZOS	223
UNK	141
FLU4	84
HEPA	30
HEP	26
PPV	26
FLUX	24
DTAPIPV	24
TDAP	23

**Table 3:** Distributions of vaccines.

VAX_NAME	Counts
COVID-19 (COVID-19 (PFIZER-BIONTECH))	5855
COVID-19 (COVID-19 (MODERNA))	5795
COVID-19 (COVID-19 (JANSSEN))	1164
ZOSTER (SHINGRIX)	165
VACCINE NOT SPECIFIED (NO BRAND NAME)	132
ZOSTER (NO BRAND NAME)	46
INFLUENZA (SEASONAL) (NO BRAND NAME)	24
INFLUENZA (SEASONAL) (FLUZONE HIGH-DOSE QUADRIVALENT)	24
INFLUENZA (SEASONAL) (FLUARIX QUADRIVALENT)	22
COVID-19 (COVID-19 (UNKNOWN))	20

**Table 4:** Distributions of vaccine manufacturers.

VAX_MANUE	Counts
PFIZER\BIONTECH	5855
MODERNA	5795
JANSSEN	1164
GLAXOSMITHKLINE BIOLOGICALS	281
UNKNOWN MANUFACTURER	247
MERCK & CO. INC.	146
SANOFI PASTEUR	93
SEQIRUS, INC.	30
NOVARTIS VACCINES AND DIAGNOSTICS	23
PFIZER\WYETH	19

**Table 5:** Distributions of symptoms.

Symptom	Counts
Headache	1596
Pyrexia	1410
Fatigue	1332
Pain	1229
Chills	1128
Pain in extremity	913
Nausea	895
Dizziness	867
Arthralgia	605
COVID-19	604

**Table 6:** Distributions of treatments.

Drug	Counts
(C0014695, Ergocalciferol)	374
(C0040165, Levothyroxine)	302
(C0004057, Aspirin)	290
(C0286651, atorvastatin)	282
(C0065374, Lisinopril)	269
(C0008318, Cholecalciferol)	268
(C0003968, Ascorbic Acid)	227
(C0025598, metFORMIN)	203
(C0025859, Metoprolol)	197
(C0028978, Omeprazole)	192

**Table 7:** Distributions of diseases.

Condition	Counts
(C0020538, Hypertensive disease)	910
(C5203670, COVID-19)	638
(C0004096, Asthma)	417
(C0041834, Erythema)	281
(C0020676, Hypothyroidism)	249
(C0042109, Urticaria)	233
(C0149931, Migraine Disorders)	226
(C0017168, Gastroesophageal reflux disease)	200
(C0011847, Diabetes)	194
(C0242350, Erectile dysfunction)	191

**Table 8:** Distributions of allergies.

Allergy	Counts
(C0030842, penicillin)	442
(C0749139, sulfa)	324
(C1705924, TAC1 wt Allele)	155
(C0009214, codeine)	148
(C0023115, latex)	131
(C0002645, amoxicillin)	114
(C0013227, Pharmaceutical Preparations)	112
(C0020517, Hypersensitivity)	111
(C0014806, erythromycin)	74
(C0026549, morphine)	66

**Table 9:** Top 10 connections of COVID-19 vaccines’ adverse effects with symptoms.

Allergy	Symptom	Count
COVID-19 (COVID-19 (PFIZER-BIONTECH))	Headache	706
COVID-19 (COVID-19 (MODERNA))	Pyrexia	663
COVID-19 (COVID-19 (MODERNA))	Headache	660
COVID-19 (COVID-19 (PFIZER-BIONTECH))	Fatigue	626
COVID-19 (COVID-19 (MODERNA))	Fatigue	586
COVID-19 (COVID-19 (PFIZER-BIONTECH))	Pyrexia	566
COVID-19 (COVID-19 (MODERNA))	Chills	565
COVID-19 (COVID-19 (MODERNA))	Pain	554
COVID-19 (COVID-19 (PFIZER-BIONTECH))	Pain	531
COVID-19 (COVID-19 (MODERNA))	Pain in extremity	459

**Table 10:** Top 10 connections of COVID-19 vaccines’ adverse effects with treatments.

Allergy	Use_Drug	Count
COVID-19 (COVID-19 (PFIZER-BIONTECH))	Ergocalciferol	169
COVID-19 (COVID-19 (MODERNA))	Ergocalciferol	165
COVID-19 (COVID-19 (MODERNA))	levothyroxine	145
COVID-19 (COVID-19 (MODERNA))	atorvastatin	139
COVID-19 (COVID-19 (MODERNA))	Cholecalciferol	132
COVID-19 (COVID-19 (PFIZER-BIONTECH))	levothyroxine	127
COVID-19 (COVID-19 (PFIZER-BIONTECH))	Cholecalciferol	114
COVID-19 (COVID-19 (PFIZER-BIONTECH))	atorvastatin	109
COVID-19 (COVID-19 (PFIZER-BIONTECH))	Ascorbic Acid	106
COVID-19 (COVID-19 (MODERNA))	Omeprazole	106

**Table 11:** Top 10 connections of COVID-19 vaccines’ adverse effects with diseases.

Allergy	Had_Condition	Count
COVID-19 (COVID-19 (PFIZER-BIONTECH))	Hypertensive disease	408
COVID-19 (COVID-19 (PFIZER-BIONTECH))	COVID-19	407
COVID-19 (COVID-19 (MODERNA))	Hypertensive disease	394
COVID-19 (COVID-19 (PFIZER-BIONTECH))	Asthma	219
COVID-19 (COVID-19 (MODERNA))	COVID-19	189
COVID-19 (COVID-19 (MODERNA))	Erythema	176
COVID-19 (COVID-19 (MODERNA))	Asthma	158
COVID-19 (COVID-19 (MODERNA))	Urticaria	122
COVID-19 (COVID-19 (PFIZER-BIONTECH))	Hypothyroidism	115
COVID-19 (COVID-19 (PFIZER-BIONTECH))	Erectile dysfunction	110

**Table 12:** Top 10 connections of COVID-19 vaccines’ adverse effects with allergies.

Allergy	Allergic_to	Count
COVID-19 (COVID-19 (MODERNA))	penicillins	706
COVID-19 (COVID-19 (PFIZER-BIONTECH))	penicillins	663
COVID-19 (COVID-19 (MODERNA))	sulfa	660
COVID-19 (COVID-19 (PFIZER-BIONTECH))	sulfa	626
COVID-19 (COVID-19 (MODERNA))	codeine	586
COVID-19 (COVID-19 (PFIZER-BIONTECH))	TAC1 wt Allele	566
COVID-19 (COVID-19 (MODERNA))	latex	565
COVID-19 (COVID-19 (MODERNA))	TAC1 wt Allele	554
COVID-19 (COVID-19 (PFIZER-BIONTECH))	Pharmaceutical Preparations	531
COVID-19 (COVID-19 (MODERNA))	amoxicillin	459

**Table 13:** Top similar symptoms related to COVID-19 vaccines. It could indicate other symptoms patients may have after receiving the vaccines.

Allergy	Symptom	Similarity
COVID-19 (COVID-19 (JANSSEN))	Tinnitus	0.267765
COVID-19 (COVID-19 (JANSSEN))	Skin lesion	0.258912
COVID-19 (COVID-19 (JANSSEN))	Thrombosis	0.253751
COVID-19 (COVID-19 (MODERNA))	Aspartate aminotransferase normal	0.243350
COVID-19 (COVID-19 (PFIZER-BIONTECH))	Diarrhea hemorrhagic	0.263173
COVID-19 (COVID-19 (PFIZER-BIONTECH))	Interchange of vaccine products	0.246813
COVID-19 (COVID-19 (UNKNOWN))	Thrombophlebitis septic	0.275156
COVID-19 (COVID-19 (UNKNOWN))	Imaging procedure	0.259467

**Table 14:** Top UMLS entity related to the COVID-19 vaccine side effects.

Vaccine	Allergic_to	Similarity
COVID-19 (COVID-19 (PFIZER-BIONTECH))	Pinus <genus>	0.230201
COVID-19 (COVID-19 (MODERNA))	Vibramycin	0.235300
**vaccine**	**had_condition**	**similarity**
COVID-19 (COVID-19 (PFIZER-BIONTECH))	Pressure Ulcer	0.236387
**vaccine**	**use_drug**	**similarity**
COVID-19 (COVID-19 (JANSSEN))	cefalexin	0.254526
COVID-19 (COVID-19 (JANSSEN))	feverfew extract	0.267852
COVID-19 (COVID-19 (PFIZER-BIONTECH))	Diclofenac	0.252584
COVID-19 (COVID-19 (MODERNA))	Vibramycin	0.235300
COVID-19 (COVID-19 (MODERNA))	Propranolol Hydrochloride	0.253414
COVID-19 (COVID-19 (MODERNA))	Combigan	0.311181
COVID-19 (COVID-19 (UNKNOWN))	Oral Tablet	0.253658
COVID-19 (COVID-19 (UNKNOWN))	Claritin-D	0.253505

**Table 15:** Machine learning Approach for prediction using GraphDB.

Model ID	Model Type	Additional Features	Positive F1-Score	Precision-Recall AUC
1	Logistic regression	Symptoms	train: 0.88 test: 0.63	train: 0.96 test: 0.65
2	XGBoost	Symptoms	train: 0.90 test: 0.67	train: 0.97 test: 0.69
3	Logistic regression	Embedding	train: 0.36 test: 0.25	train: 0.33 test: 0.23
4	XGBoost	Embedding	train: 0.58 test: 0.31	train: 0.91 test: 0.24
5	XGBoost	Symptoms + Embedding	train: 0.90 test: 0.67	train: 0.97 test: 0.69

## Data Availability

The dataset used in the current study will be made available on request.

## Supplementary Materials

The following supporting information can be downloaded at: https://www.mdpi.com/article/10.3390/healthcare10081419/s1, Table S1: Subset of VAERSDATA Fields; Table S2: VAERSVAX Fields; Table S3: VAERSSYMPTOMS Fields; Table S4: Nodes and edges abstraction.
