# Supplementary material for: Modeling COVID-19 Vaccine Adverse Effects with a Visualized Knowledge Graph Database
Source: Healthcare (Basel). 2022 Jul 29;10(8):1419. doi: 10.3390/healthcare10081419 (PMC9407998; doi:10.3390/healthcare10081419)
Supplement: Supplementary file 1 [file healthcare-10-01419-s001.zip › healthcare-1823471-supplementary.pdf]

Supplementary Materials

Table S1. Subset of VAERSDATA Fields. source: https://vaers.hhs.gov/data.html

Header	Type	VAERS 2 Form	VAERS 1 Form	Description of Contents
VAERS_ID	Num (7)	Not derived	Not derived	VAERS identification number
RECVDATE	Date	Not derived	Not derived	Date report was received
STATE	Char (2)	Derived	Box 1	State
AGE_YRS	Num (xxx.x)	Item 6	Box 4	Age in years
CAGE_YR	Num (xxx)	Derived	Derived	Calculated age of patient in years
CAGE_MO	Num (x.x)	Derived	Derived	Calculated age of patient in months
SEX	Char (1)	Item 3	Box 5	Sex
RPT_DATE	Date	Discontinued	Box 6	Date form completed
SYMPTOM_TEXT	Char (32,000)	Item 18	Box 7	Reported symptom text

Table S2. VAERSVAX Fields. source: https://vaers.hhs.gov/data.html

Header	Type	Description de Contents
VAERS_ID	Num (7)	VAERS identification number
VAX_TYPE	Char (15)	Administered vaccine type
VAX_MANU	Char (40)	Vaccine manufacturer
VAX_LOT	Char (15)	Manufacturer's vaccine lot
VAX_DOSE_SERIES	Char (3)	Number of doses administered
VAX_ROUTE	Char (6)	Vaccination route
VAX_SITE	Char (6)	Vaccination site
VAX_NAME	Char (100)	Vaccination name

Table S3. VAERSSYMPTOMS Fields. source: https://vaers.hhs.gov/data.html

Heading	Type	Description de Contents
VAERS_ID	Num (7)	VAERS identification number
SYMPTOM1	Char (100)	Adverse event MedDRA term 1
SYMPTOMVERSION1	Num (xx.xx)	MedDRA dictionary version 1
SYMPTOM2	Char (100)	Adverse event MedDRA term 2
SYMPTOMVERSION2	Num (xx.xx)	MedDRA dictionary version 2
SYMPTOM3	Char (100)	Adverse event MedDRA term 3
SYMPTOMVERSION3	Num (xx.xx)	MedDRA dictionary version 3
SYMPTOM4	Char (100)	Adverse event MedDRA term 4
SYMPTOMVERSION4	Num (xx.xx)	MedDRA dictionary version 4
SYMPTOM5	Char (100)	Adverse event MedDRA term 5
SYMPTOMVERSION5	Num (xx.xx)	MedDRA dictionary version 5

Table S4. Nodes and edges abstraction

Nodes	Explain	Property	Relations
Patient	Patient vaccination record	VAERS_ID, RECVDATE, STATE, AGE_YRS, SEX, DIED, L_THREAT, DISABLE, VAX_DATE, ONSET_DATE, NUMDAYS	TOOK_VACCINE: patient -> Vaccine; HAD_SYMPTOM: patient -> Symptom; USED_DRUG (derived): patient -> UMLS; HAD_CONDITION (derived): patient -> UMLS; ALLERGIC_TO (derived): patient -> UMLS
Vaccine	Vaccine information	VAX_NAME, VAX_MANU, VAX_TYPE	
Symptom	Side effect symptoms	SYMPTOM, SYMPTOMVERSION	
UMLS [1]	Extracted UMLS entity representing medical/condition/allergy history	CUI, name	

[1] derived Nodes and Edges are extracted from unstructured texts.
